# Medical Students’ Demographic Characteristics and Their Perceptions of Faculty Role Modeling of Respect for Diversity

**DOI:** 10.1001/jamanetworkopen.2021.12795

**Published:** 2021-06-04

**Authors:** Jasmine Weiss, Lilanthi Balasuriya, Laura D. Cramer, Marcella Nunez-Smith, Inginia Genao, Rosana Gonzalez-Colaso, Ambrose H. Wong, Elizabeth A. Samuels, Darin Latimore, Dowin Boatright, Mona Sharifi

**Affiliations:** 1 National Clinician Scholars Program, Yale School of Medicine, New Haven, Connecticut; 2 Department of Internal Medicine, Yale School of Medicine, New Haven, Connecticut; 3 Department of Emergency Medicine, Yale School of Medicine, New Haven, Connecticut; 4 Department of Emergency Medicine, The Warren Alpert Medical School, Brown University, Providence, Rhode Island; 5 Department of Pediatrics, Yale School of Medicine, New Haven, Connecticut

## Abstract

**Importance:**

Faculty role modeling is critical to medical students’ professional development to provide culturally adept, patient-centered care. However, little is known about students’ perceptions of faculty role modeling of respect for diversity.

**Objective:**

To examine whether variation exists in medical students’ perceptions of faculty role modeling of respect for diversity by student demographic characteristics.

**Design, Setting, and Participants:**

This cross-sectional study analyzed data from the Association of American Medical Colleges’ 2016 and 2017 Medical School Graduation Questionnaire, which was administered to graduating students at 140 accredited allopathic US medical schools. Data were analyzed from January 1 to November 1, 2020.

**Main Outcomes and Measures:**

Students’ perceptions of faculty role modeling of respect for diversity by the independent variables sex, race/ethnicity, sexual orientation, and age. Multivariable logistic regression was used to examine the extent to which student-reported perceptions of faculty respect for diversity varied by demographic characteristics, and logistic regression models were sequentially adjusted first for demographic characteristics and then for marital status and financial variables.

**Results:**

Of 30 651 students who completed the survey, the final study sample consisted of 28 778 respondents, representing 75.4% of the 38 160 total US medical school graduates in 2016 and 2017. Of the respondents, 14 804 (51.4%) were male participants and 1506 (5.2%) identified as lesbian, gay, or bisexual (LGB); a total of 11 926 respondents (41.4%) were 26 years or younger. A total of 17 159 respondents (59.6%) identified as White, 5958 (20.7%) as Asian, 1469 (5.1%) as Black/African American, 2431 (8.4%) as Hispanic/Latinx, and 87 (0.3%) as American Indian/Alaska Native/Native Hawaiian/Pacific Islander individuals. Overall, 5101 students (17.7%) reported perceiving that faculty showed a lack of respect for diversity. Of those who identified as Black/African American students, 540 (36.8%) reported perceiving a lack of faculty respect for diversity compared with 2468 White students (14.4%), with an OR of perceived lack of respect of 3.24 (95% CI, 2.86-3.66) after adjusting for other demographic characteristics and covariates. American Indian/Alaska Native/Native Hawaiian/Pacific Islander (OR, 1.73; 95% CI, 1.03-2.92), Asian (OR, 1.62; 95% CI, 1.49-1.75), or Hispanic/Latinx (OR, 1.43; 95% CI, 1.26-1.75) students also had greater odds of perceiving a lack of faculty respect for diversity compared with White students. Female students had greater odds compared with male students (OR, 1.17; 95% CI, 1.10-1.25), and students who identified as LGB (OR, 1.96; 95% CI, 1.74-2.22) or unknown sexual orientation (OR, 1.79; 95% CI, 1.29-2.47) had greater odds compared with heterosexual students. Students aged 33 years or older had greater odds of reporting a perceived lack of respect compared with students aged 26 years or younger (OR, 1.81; 95% CI, 1.58-2.08).

**Conclusions and Relevance:**

In this cross-sectional study, female students, students belonging to racial/ethnic minority groups, and LGB students disproportionately reported perceiving a lack of respect for diversity among faculty, which has important implications for patient care, the learning environment, and the well-being of medical trainees.

## Key Points

**Question:**

Do medical students’ perceptions of faculty role modeling of respect for diversity vary by students’ demographic characteristics?

**Findings:**

In this cross-sectional study of 28 778 graduating medical students, 17.7% of students reported perceiving that faculty showed a lack of respect for diversity. Female students, students belonging to racial/ethnic minority groups, and lesbian, gay, or bisexual students disproportionately reported perceiving a lack of respect for diversity among faculty.

**Meaning:**

The findings suggest that faculty role modeling of respect for diversity is perceived differently by students from marginalized groups.

## Teaser

This cross-sectional study uses data from the Association of American Medical Colleges’ 2016 and 2017 Medical School Graduation Questionnaire to examine whether medical students’ perceptions of faculty respect for diversity differ by students’ sex, race/ethnicity, sexual orientation, and age.

## Introduction

Medical schools aim to educate compassionate and culturally sensitive physicians to care for patients from diverse backgrounds. This education occurs formally through integrated cultural competency curricula and informally through role modeling by faculty, residents, and other clinical instructors.^1,2^ The behaviors displayed by faculty may be associated with students’ attitudes and behaviors and are considered a part of what is called the hidden curriculum.^3^ For example, exposure to negative comments about African American patients is associated with higher levels of implicit and explicit racial biases among medical students.^4,5^ One study^6^ found that students who witnessed discriminatory comments by faculty toward lesbian, gay, bisexual, or transgender (LGBT) patients showed greater levels of implicit bias toward sexual and gender minority populations. Faculty role modeling is widely acknowledged as an integral aspect of professional development toward culturally adept, patient-centered care.^7^

Although prior literature has examined associations between overall faculty role modeling and student behaviors and student well-being,^4,5,8^ little is known about medical students’ perceptions of faculty role modeling of respect for diversity. To our knowledge, the current study is unique in using a nationally representative sample of US students to examine perceptions of faculty role modeling of respect for diversity and whether these perceptions vary among students with different demographic characteristics. Data on these perceptions may be useful for accrediting bodies, such as the Liaison Committee for Medical Education and the Accreditation Council for Graduate Medical Education, which set standards for programs to promote the retention, recruitment, and inclusion of diverse trainees, administrators, and faculty.^9,10^ Understanding how faculty role modeling of respect for diversity impacts students from diverse backgrounds is important for a safe and inclusive learning environment.

Each year, the Association of American Medical Colleges (AAMC) administers a survey to graduating medical students that includes questions about faculty role modeling. In this study, our aims were to (1) describe the perceptions of faculty respect for diversity among a national sample of medical students and (2) assess whether students’ perceptions of faculty role modeling of respect for diversity varied by students’ demographic characteristics. We hypothesized that students from historically marginalized groups (female individuals, racial/ethnic minority populations, and LGB individuals) and older students with the potential for greater cumulative exposure to discrimination^11,12^ would be more likely to have a negative perception of whether faculty show respect for diversity.

## Methods

### Sample

In this cross-sectional study, we analyzed survey data from the 2016 and 2017 AAMC Medical School Graduation Questionnaire (GQ). The GQ is given each spring to graduates from allopathic US medical schools accredited by the Liaison Committee for Medical Education and includes questions about demographic characteristics, clinical experiences, finances, career plans, and the learning environment. Of the 38 160 US medical school graduates in 2016 and 2017, 30 841 (80.8%) completed the questionnaire. Medical schools that did not have graduating classes in both 2016 and 2017 were excluded from the data set. We further excluded students with a missing response to the survey item addressing faculty role modeling of respect for diversity. Data were analyzed from January 1 to November 1, 2020. This study was deemed exempt from review by the Yale Human Research Protection Program because the study involved nonhuman participant research and data were deidentified. The study followed the Strengthening the Reporting of Observational Studies in Epidemiology (STROBE) reporting guideline.^13^

### Measures

#### Student Perceptions of Faculty Role Modeling of Respect for Diversity

The GQ assessed students’ perceptions of the extent to which faculty members were role models for respect for diversity with the question, “Please rate how often the following professional behaviors/attitudes are [were] demonstrated by your medical school’s faculty: Respecting diversity.” Responses were measured on a 6-point Likert scale, with 1 indicating never; 2, almost never; 3, sometimes, 4, fairly often; 5, very often; and 6, always. To explore the extent to which student reports of faculty respect for diversity may have differed by student demographic characteristics, we dichotomized the responses into 2 categories: perceived lack of respect (never, almost never, sometimes, or fairly often) and perceived respect (very often or always). A priori, we considered the response fairly often as indicating a perceived lack of respect.

#### Demographic Variables

Students’ responses from the GQ were linked to self-reported demographic data via AAMC data applications and services.^14^ Demographic variables included sex (male vs female), race/ethnicity, sexual orientation (heterosexual, LGB, or unknown), and age. Given the small number of students self-reporting as transgender and consequent privacy concerns, gender identity data were not available to the study team.^15^

The AAMC GQ collects race and ethnicity data in a single question. This allows students to select multiple race/ethnicity options, including Hispanic ethnicity.^16,17^ First, we defined Hispanic/Latinx individuals as those who selected items from the Hispanic, Latino, or of Spanish origin category irrespective of individuals who selected additional race/ethnicity options. The remaining students not categorized as Hispanic/Latinx individuals were categorized as Black/African American, Asian, White, unknown, or other individuals. We combined American Indian, Alaskan Native, tribal affiliation, Native Hawaiian, and Pacific Islander into 1 category given the small number of students in these categories. Students categorized as other individuals did not self-identify with any of the subcategories of race/ethnicity provided as selection options. We categorized students who reported multiple races/ethnicities as multiracial–underrepresented in medicine vs multiracial–not underrepresented in medicine based on the presence or absence of at least 1 of the following selections: Black/African American, American Indian, Alaskan Native, tribal affiliation, Native Hawaiian, and Pacific Islander.

Students reported their age based on fixed categories: younger than 24 years, 24 to 26 years, 27 to 29 years, 30 to 32 years, or 33 years or older. Given the small number of students 26 years or younger, we combined these students into 1 age category.

#### Covariates

Prior studies^18,19,20,21^ suggested that marital status and financial concerns are associated with personal distress among medical students, which may influence perceptions. Therefore, we included marital status (unknown, single, legally married, common law or civil union, divorced, separated, or widowed) and financial considerations (scholarships [yes or no] and loans [yes or no]) as covariates.

### Statistical Analysis

We calculated descriptive statistics for the demographic variables using proportions because all of the variables were categorical. Of the students excluded owing to a missing response to the faculty respect for diversity item, we used Pearson χ^2^ tests (or Fisher exact tests when appropriate) to compare their demographic characteristics with those of students included in the study sample.

Using multivariable logistic regression, we examined the extent to which student-reported perceptions of faculty respect for diversity varied by demographic characteristics and sequentially adjusted all logistic regression models first for demographic characteristics and then for marital status and financial variables. We completed a sensitivity analysis to assess the impact of grouping the fairly often responses with the very often or always responses for the outcome variable of the logistic regression model. To assess multicollinearity of the independent variables, we examined the variance inflation factor. We used the Hosmer-Lemeshow test to assess model fit. We included separate interaction terms for sex, race/ethnicity, sexual orientation, and age to examine effect modification of any observed association between perceived respect and demographic characteristics.

We present the associations as odds ratios (ORs) with 95% CIs. All *P* values were from 2-sided tests. Results were considered statistically significant at *P* < .05. We performed all data analyses using Stata/SE, version 16.1 (StataCorp LLC).

## Results

Of 30 651 total survey responses, the final study sample consisted of responses from 28 778 students, representing 75.4% of the total 38 160 US medical school graduates in 2016 and 2017. Of the respondents, 14 804 (51.4%) were male students and 13 974 (48.6%) were female students; 17 159 (59.6%) were White, 5958 (20.7%) Asian, 1469 (5.1%) Black/African American, 2431 (8.4%) Hispanic/Latinx, and 87 (0.3%) American Indian/Alaska Native/Native Hawaiian/Pacific Islander individuals. A total of 11 926 respondents (41.4%) were aged 26 years or younger and 1668 (5.8%) were 33 years or older at the time of completion of the survey. A total of 1506 respondents (5.2%) identified as LGB. Demographic characteristics of respondents are given in Table 1.

**Table 1.** Demographic Characteristics of the Study Population

Characteristic	Participants, No. (%) (N = 28 778)^**a**^
Sex	
Male	14 804 (51.4)
Female	13 974 (48.6)
Race/ethnicity	
White	17 159 (59.6)
Asian	5958 (20.7)
Black/African American	1469 (5.1)
Hispanic/Latinx	2431 (8.4)
American Indian/Alaska Native/Native Hawaiian/Pacific Islander	87 (0.3)
Multiracial, not URiM	745 (2.6)
Multiracial, URiM	357 (1.2)
Other	285 (1.0)
Unknown	287 (1.0)
Sexual orientation	
Heterosexual	26 252 (91.2)
LGB	1506 (5.2)
Unknown	1020 (3.5)
Age, y	
≤26	11 926 (41.4)
27-29	11 865 (41.2)
30-32	3319 (11.5)
≥33	1668 (5.8)

Abbreviations: LGB, lesbian, gay, or bisexual; URiM, underrepresented in medicine.

^a^ Percentages may not sum to 100 owing to rounding.

We excluded 1873 students (6.1%) who did not answer the item about faculty respect for diversity. Those excluded for missing responses were more likely to be male than female (1062 [56.7%] vs 811 [43.3%]; *P* < .001), to identify as Black/African American or Asian than White (165 [10.1%] vs 456 [7.1%] vs 988 [5.4%]; *P* < .001), to identify as unknown sexual orientation than heterosexual (1747 [63.1%] vs 118 [0.5%]; *P* < .001), and to be older (30-32 years vs ≤26 years: 257 [7.2%] vs 206 [5.7%]; *P* < .009).

**Figure 1.**
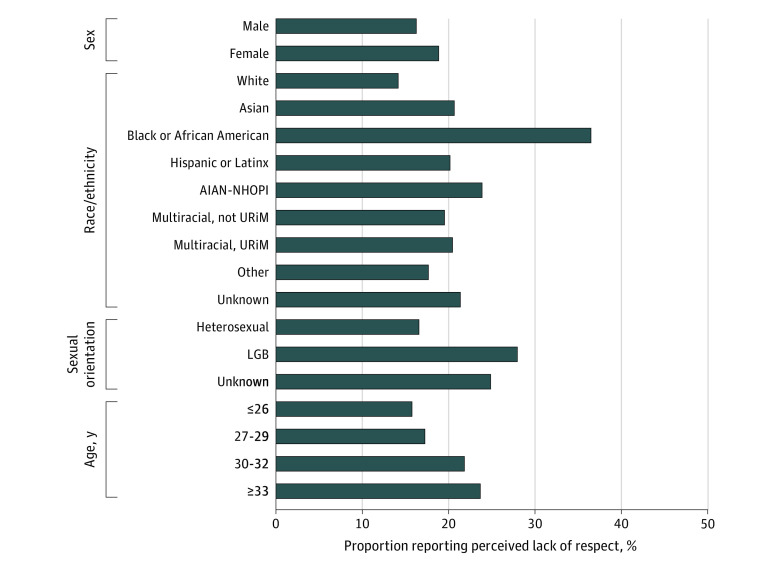
Perceived Lack of Respect by Demographic Characteristics For all comparisons, *P* < .001. AIAN-NHOPI indicates American Indian/Alaska Native/Native Hawaiian/Pacific Islander; LGB, lesbian, gay, or bisexual; and URiM, underrepresented in medicine.

In the study sample, 5101 respondents (17.7%) reported a perceived lack of respect for diversity among faculty. In response to how often respect for diversity was demonstrated by the medical school’s faculty, 40 (0.1%) responded never; 132 (0.5%), almost never; 1283 (4.5%), sometimes; and 3646 (12.7%), fairly often. A total of 12 102 respondents (42.2%) rated faculty as very often respectful and 11 575 (40.4%) as always respectful. The prevalence of perceived lack of respect by demographic characteristics is shown in Figure 1. The results of logistic regression models adjusted for remaining demographic characteristics and covariates are given in Table 2. The variance inflation factor remained at a reasonable level for each of the independent variables, the highest level (1.1) being for marital status. The result of the Hosmer-Lemeshow test was not significant, indicating good model fit.

**Table 2.** Perceived Lack of Respect for Diversity by Demographic Characteristics

Characteristic	OR (95% CI)	
	Unadjusted	Adjusted^a^
Sex		
Male	1 [Reference]	1 [Reference]
Female	1.20 (1.13-1.28)	1.17 (1.10-1.25)^b^
Race/ethnicity		
White	1 [Reference]	1 [Reference]
Asian	1.58 (1.46-1.70)^b^	1.62 (1.49-1.75)^b^
Black/African American	3.50 (3.10-3.94)^b^	3.24 (2.86-3.66)^b^
Hispanic/Latinx	1.57 (1.34-1.85)^b^	1.43 (1.26-1.75)^b^
American Indian/Alaska Native/Native Hawaiian/Pacific Islander	1.94 (1.17-3.21)^c^	1.73 (1.03-2.92)^c^
Multiracial, not URiM	1.54 (1.28-1.86)^b^	1.45 (1.20-1.76)^b^
Multiracial, URiM	1.50 (1.32-1.71)^b^	1.58 (1.26-1.63)^b^
Other	1.31 (0.95-1.74)	1.25 (0.90-1.74)
Unknown	1.64 (1.22-2.20)^b^	1.73 (1.29-2.33)^b^
Sexual orientation		
Heterosexual	1 [Reference]	1 [Reference]
LGB	1.94 (1.72-2.18)^b^	1.96 (1.74-2.22)^b^
Unknown	1.95 (1.60-2.38)^b^	1.79 (1.29-2.47)^b^
Age, y		
≤26	1 [Reference]	1 [Reference]
27-29	1.12 (1.05-1.21)^b^	1.15 (1.07-1.24)^b^
30-32	1.50 (1.36-1.66)^b^	1.61 (1.45-1.79)^b^
≥33	1.66 (1.46-1.88)^b^	1.81 (1.58-2.08)^b^
Marital status		
Single	NA	1 [Reference]
Legally married	NA	0.81 (0.75-0.88)^b^
Common law or civil union	NA	1.56 (1.03-2.37)
Divorced	NA	1.10 (0.81-1.49)
Separated	NA	0.88 (0.42-1.85)
Widowed	NA	0.77 (0.16-3.63)
Unknown	NA	1.45 (0.84-2.51)
Scholarship		
No	NA	1 [Reference]
Yes	NA	1.02 (0.96-1.10)
Student loans		
No	NA	1 [Reference]
Yes	NA	1.08 (1.00-1.16)

Abbreviations: LGB, lesbian, gay, or bisexual; NA, not applicable; OR, odds ratio; URiM, underrepresented in medicine.

^a^ Adjusted for remaining demographic characteristics including race/ethnicity, sexual orientation, age, sex, and financial status.

^b^ *P* < .001.

^c^ *P* < .05.

In total, 540 of 1469 Black/African American students (36.8%) reported perceiving a lack of respect for diversity among faculty compared with 2468 of 17 159 White students (14.4%). The odds of perceived lack of respect were 3-fold greater among Black/African American students than among White students (OR, 3.24; 95% CI, 2.86-3.66). We also observed greater odds of perceived lack of faculty respect for diversity among those who identified as American Indian/Alaska Native/Native Hawaiian/Pacific Islander (OR, 1.73; 95% CI, 1.03-2.92), Asian (OR, 1.62; 95% CI, 1.49-1.75), or Hispanic/Latinx (OR, 1.43; 95% CI, 1.26-1.75) students compared with White students. In the sensitivity analysis after adjusting the definition of faculty’s respect for diversity to include responses indicating that respect was perceived fairly often, Black/African American students had even greater odds of perceived lack of respect compared with White students (OR, 5.04; 95% CI, 4.21-6.03).

Compared with male students, female students had greater odds of reporting a lack of respect for diversity among faculty (OR, 1.17; 95% CI, 1.10-1.25), as did students who identified as LGB (OR, 1.96; 95% CI, 1.74-2.22) or as unknown (OR, 1.79; 95% CI, 1.29-2.47) with respect to sexual orientation compared with students who identified as heterosexual. Students aged 33 years or older had greater odds of reporting a perceived lack of respect compared with students 26 years or younger (OR, 1.81; 95% CI, 1.58-2.08) after adjusting for other demographic characteristics and covariates (Table 2).

**Figure 2.**
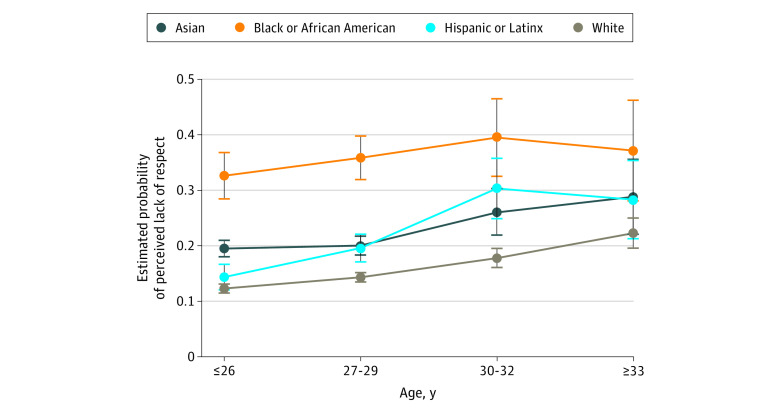
Association Between Perceived Lack of Respect and Age by Race/Ethnicity Whiskers indicate 95% CIs.

We observed a significant interaction between race/ethnicity and age but not between other demographic variables. The results for Black/African American, Asian, White, and Hispanic/Latinx students are presented in Figure 2. Black/African American students had the greatest probability of reporting a perceived lack of faculty respect in each subcategory compared with their age-matched counterparts.

## Discussion

In this cross-sectional study, 17.7% of medical students in the sample reported perceiving a lack of respect for diversity among faculty. Medical students who identified as members of racial/ethnic minority populations or who self-reported being female, an LGB individual, or an older student had higher odds of reporting that faculty lacked respect for diversity compared with their majority-population counterparts. The association between race/ethnicity and perceived lack of respect for diversity differed by age, with the highest probability of perceiving lack of respect observed among Black/African American students in each age subcategory compared with their age-matched counterparts.

Our observation that a perceived lack of respect was prevalent among medical students may reflect students’ perceptions regarding faculty-patient interactions, their experiences within the overall learning environment, and the direct social interaction or lack thereof between students and faculty. During each clinical encounter in a teaching context, faculty should exemplify respectful interpersonal communication and effective patient engagement despite differences in culture, beliefs, and background. Gaps in patient-provider communication may be associated with increased health disparities and higher levels of bias toward female patients,^22^ LGB individuals,^23^ and patients from racial/ethnic minority populations.^22,23,24,25^

This study’s findings that medical students who identified as being Black/African American, Hispanic/Latinx, American Indian/Alaska Native/Native Hawaiian/Pacific Islander, female, LGB, or older more often reported perceiving a lack of faculty respect for diversity may be attributable to differences in perceptions of biased patient-provider interactions. As described by critical race theory,^26^ students of minoritized racial/ethnic backgrounds may exhibit greater consciousness of race/ethnicity given their lived experiences and may be more acutely aware of discrimination in the patient care setting. Similarly, female students may witness differential treatment of male and female patients who have similar clinical presentations.^22,27,28^ Students who identify as LGB also witness biased behaviors, such as inappropriate humor and discriminatory comments toward patients who are LGB.^6^

Faculty may be unaware that they are modeling negative behaviors and may regard themselves as nonbiased; however, they may demonstrate negative unconscious attitudes and biases, such as aversive racism and gender blindness, toward marginalized groups during patient encounters.^27,29,30,31^ Students may also feel unsupported in situations in which patients demonstrate racism, sexism, or discrimination toward LGB trainees and faculty do not speak out against this behavior.^32^

A lack of respect for diversity can be evident during specific patient encounters and in the overall learning environment. During the preclinical years, race is often taught as a biological characteristic as opposed to racism being taught as a social construct that is associated with health outcomes.^33,34^ The findings of the present study support prior literature indicating that negative faculty behavior and an unprofessional culture are part of the hidden curriculum^3^ that can contribute to a more negative institutional climate, particularly for members of racial/ethnic minority populations.^8^ For marginalized groups, a greater prevalence of the perceived lack of faculty respect for diversity may contribute to a more negative institutional psychological climate through the perception of increased discrimination and racial tension.^35^ In the present study, Black/African American students had the highest prevalence among all racial/ethnic groups of perceiving that faculty role models demonstrated a lack of respect. This result supports previous findings that Black students may have negative perceptions of the medical school environment, specifically when interacting with White faculty.^36^

A lack of respect for diversity in the learning environment also may be more readily perceived by female and LGB students. During preclinical years, studies have demonstrated that women are underrepresented in case discussions, and sexual orientation is often presented as a risk factor for specific diseases.^37^ Prior literature has shown that female medical students experience patriarchal views about women in medicine through sexist comments and the use of inappropriate language.^38^ LGB students may be exposed to discriminatory comments and may witness these comments toward or refusal of care for LGB, transgender, and gender nonbinary patients.^39^ These behaviors can lead to the perception of decreased learning opportunities and a lack of support and mentorship, which serve as barriers to academic success^40^ and may undermine institutional recruitment and retention efforts.^9,10,41^

In addition to indirect observations of faculty interactions with patients and colleagues, marginalized students may perceive a lack of respect directed toward them in the form of microaggressions, discrimination, mistreatment, and sexual harassment. Racial/ethnic microaggressions perpetuated by faculty can cause students to question and ruminate on the offensive nature of subtle remarks, resulting in undue stress and distraction that are associated with a decline in academic performance.^42^ Discrimination toward marginalized groups may be associated with students’ choice of specialty and the decision to care for minoritized patients.^38,43^ Studies have shown that observing negative comments by faculty is associated with higher levels of implicit and explicit bias in trainees that has even been noted early during the medical school interview process.^4,5,44^

This study’s findings suggest that faculty should consider how their interpersonal interactions with trainees in the learning environment can adversely affect students from marginalized groups. Qualitative and mixed-methods approaches may be used to further understand perceptions of diversity from both the student and the faculty points of view and to design interventions that modify the systemic and interpersonal factors influencing these perceptions. This study also suggests the need for more robust faculty development that promotes inclusivity and emphasizes training on bias, discrimination, microaggressions, and respect for diversity.

As a part of the sample selection, Black/African American students and those who identified as of unknown sexual orientation were less likely to answer the survey item about faculty role modeling of respect for diversity. This may have been attributable to sentiments of distrust or fear of retribution among marginalized students when reporting faculty behavior. In the future, the AAMC should consider strategies to reassure students that such data will be collected anonymously and should consider how psychological safety can be maintained.

Medical schools and faculty, who are leaders in academia, and medical school accrediting bodies should reaffirm their approach to creating an inclusive environment. We believe that open, frequent dialogue is needed to discuss the effects of structural racism, sexism, and discrimination toward LGB individuals in medical education and patient care to foster a culture that is more respectful of diversity.

Robust systems can be implemented for trainees, staff, and faculty to report discrimination in a safe and anonymous way. We believe that individuals who perpetrate negative behaviors should be held accountable for their actions and provided with strategies to help mitigate their personal biases to facilitate a more inclusive teaching environment. As governing bodies such as the Accreditation Council for Graduate Medical Education and the Liaison Committee for Medical Education continue to implement diversity standards, refining ways to measure inclusivity within the learning environment should also be a priority.

### Strengths and Limitations

This study has strengths. To our knowledge, this is the first study that assesses faculty role modeling of respect for diversity by demographic characteristics. A large and nationally representative sample of US medical students was included in the analysis.

This study also has limitations. The cross-sectional survey design allowed us to examine the association between demographic characteristics and students’ perceptions of respect for diversity among faculty only at a single time point. This study’s findings may also be subject to recall bias because the GQ is completed at the time of graduation, whereas students’ experiences could have occurred at any point during training. Although we adjusted for marital status and financial considerations, other, unmeasured confounders such as primary language, religious beliefs, and immigrant status may have influenced the associations found in this study. In addition, this study’s measure of respect for diversity was broad and subjective. Although there was a significant interaction between race/ethnicity and age, the ability to detect intersectionality related to race/ethnicity and sex or LGB status may have been limited given the sample size within some race categories. The study also may have captured many aspects of faculty role modeling in interactions that faculty had with patients, peers, and students. Despite these limitations, the descriptive observations of this study merit further evaluation.

## Conclusions

In this cross-sectional study, female students, students from racial/ethnic minority groups, and LGB medical students disproportionately perceived a lack of respect for diversity from faculty. This perceived lack of respect for diversity has important implications for patient care, the learning environment, and social interactions with trainees. Further studies are needed to assess the mediators of students’ perceptions of faculty respect for diversity and how these perceptions may more directly impact student well-being as well as to identify optimal interventions to improve role modeling of respect for diversity among faculty.
